# The assessment of the related factors of emotional divorce among Iranian people during the Covid-19 pandemic: a descriptive study

**DOI:** 10.1186/s40359-023-01395-w

**Published:** 2023-10-27

**Authors:** Zahra Sadeghzadeh, Robabeh Ghodssi-ghassemabad, Mostafa Hamdieh, Samirasadat Shariatpanahi, Faeze Babazadeh, Mitra Abdoli, Keshvar Samadaee Gelehkolaee

**Affiliations:** 1 Men’s Health and Reproductive Health Research Center, Shahid Beheshti University of Medical Sciences, Tehran, Iran; 2 Psychosomatic department, Shahid Beheshti University of medical sciences, Tehran, Iran; 3 Sexual and Reproductive Health Research Center, Department of Reproductive Health and Midwifery, Faculty of Nursing and Midwifery, Mazandaran University of Medical Sciences, Sari, Iran

**Keywords:** Emotion, Divorce, Covid-19, Iranian people

## Abstract

**Background:**

When a couple experiences emotional divorce, it can lead to boredom, a decrease in their connection, feelings of sadness and despair, and reduced reliance on one another. These consequences can have a lasting impact on the entire family. Therefore, the present study was designed to assess the affecting related factors of emotional divorce among Iranian people during the Covid19 pandemic.

**Methods:**

A descriptive-analytical study was performed on 900 men and women from 22 districts of Tehran who were selected by the available sampling methods from March to October 2021. Data collection tools are Guttmann demographic and Emotional Divorce Questionnaires. The Questionnaires were completed by the participants of the study. R 4.0.2 software was used to analyze the data, in addition, an independent t-test and chi-square were used to compare the subjects in terms of emotional divorce. Also, the multiple logistic regression method was used to determine the independent factors affecting emotional divorce.

**Results:**

The results showed that the related factors of emotional divorce include age, marriage rank, and duration of the marriage, choosing the spouse by parents, the education level of the couple, the job of the couple, infection of Covid19, and having disputes before Covid19 infection outbreak.

**Conclusion:**

Emotional divorce is considered as a social harm which could be the prelude to legal divorce. Therefore, studying affecting factors in any society can pave the way for culture-based interventions to reduce such social harms.

**Supplementary Information:**

The online version contains supplementary material available at 10.1186/s40359-023-01395-w.

## Background

Family breakdown is a significant social issue, and emotional divorce is a crucial factor in this phenomenon. It can lead to boredom, cooling of relationships, feelings of sadness and despair, and the couple’s independence from each other [1]. Before the legal divorce, emotional divorce can seep into a relationship between couples and gradually harm the family unit. It creates distance between the partners, leading to feelings of animosity, coldness, loneliness, and a lack of friendship, which can create a negative atmosphere in the family. Finally, positions held by couples in the decision-making for separation/legal divorce are chosen: initiators, non-initiators, spokesperson initiators, and active non-initiators [2].

A 2020 study found that the worldwide divorce rate in 2017 was approximately 44% [3]. In Doherty’s study in 2021 according to the Iranian Civil Registration (2016), Iran’s divorce rate was 2.8 times over 10 years [4]. According to a study, Iranian nurses had an emotional divorce rate of 7.6% [5]. In addition, we can mention a bigger tragedy, called emotional divorce, which makes family Cohesion unpleasant and inappropriate, destroys the friendship and love between couples, and results in weakness and instability in families [6]. Emotional divorce can cause a Lack of happiness, anxiety, depression, and feelings of inferiority, as well as physical and mental illness in families. Finally, negative consequences for the children of these families, such as isolation, academic failure, and aggression will appear [7].

Since the outbreak of COVID-19 and subsequent quarantines to prevent and control the spread of the virus, couples dealing with emotional divorce have entered a new phase of conflicts. These conflicts include economic and livelihood recession, financial loss, and shame [8, 9]. Experiencing negative emotions, anxiety, fear of disease transmission [10], sleep disorders, post-traumatic stress disorder (PTSD), depression [11], decreasing of religious, recreational, and cultural activities [12], decreasing of physical activity [9] were the most important factors, which was imposed on families. Several qualitative studies have explained the harmful psychological effects of the Covid-19 epidemic on Iranian families, including negative emotions induced by the situation [10, 13].

The emotional divorce between couples along with the negative consequences due to the Covid-19 outbreak particularly make negative changes in the lifestyle of couples and had a direct impact on their quality of life and lifestyle [14].

Based on the evidence, due to the social harms during this difficult period and the increasing rate of emotional divorce [15], this study was designed with the goal of the assessment of the related factors of emotional divorce among Iranian people during the Covid-19 Pandemic.

## Materials and methods

The present study is a descriptive-analytical cross-sectional study. 900 men and women were selected by the available sampling method from March to October 2021 from 22 districts of Tehran and entered the study. The infection of Covid-19 in Iran was officially announced in February 2019 and it was in the second lockdown until the sample collection date of the present project in October 2021. After receiving the code of ethics from the Ethics Committee of the University of Medical Sciences, the initial interview was conducted by a clinical psychologist to diagnose sexual and psychological disorders. Data were collected from the target group in the locations such as schools, hospitals, offices, banks, and grocery stores. The psychologist explained the objectives of the study and emphasized the confidentiality of their information. After checking that the inclusion criteria were met, the subjects were provided with the questionnaire. These criteria consist of being literate, having been married for at least one year, and currently cohabiting.

Exclusion criteria include unwillingness to participate, couples about to separate, having a history of sexual dysfunction, psychiatric disorders such as depression, or using neuroleptics during the previous month (self-report). The sample size is estimated to be 4,705 due to the prevalence of 11.7% of emotional divorce in another study in Iran (considering the acceptable difference of 3.4% of the prevalence of the sample size of 4,705 people, which was appraised at 20% of non-cooperation and loss) [16].

Data collection tools included the Demographic Questionnaire and the Guttman Emotional Divorce Questionnaire, which were furnished by the involved couples. The demographic questionnaire included 20 questions about age, gender, type of employment, level of education, type of residence, the person with a family disability, covid19 infection, type of companionship, and economic status. The Emotional Divorce Questionnaire was designed and developed by Gottman in 1994 [17]. It has 24 questions and must be answered in a yes, no way. “Yes” got one point and “No” got zero. The range of scale scores is between 0 and 24. The higher the number of “yes” answers, the greater the likelihood of an emotional divorce, and the higher the number of “no” answers, the fewer the likelihood of an emotional divorce. The method of calculating the score is that the scores obtained from the aggregation of options are multiplied by the number of questionnaires. In the interpretation of scores, a score between 0 and 8 is a weak separation probability, a score between 8 and 16 is a moderate separation probability and a score above 16 is a strong separation probability. According to the study conducted by Musavi et al., it was found that the questionnaire had both face and construct validity, which suggests that the content was valid [18]. In the study of Ahmadi et al., The reliability of the questionnaire with Cronbach’s alpha was reported to be 89% [19].

### Statistical analysis

According to the 11.7% prevalence of emotional divorce in people with non-academic education in Iran [16], and considering the acceptable difference of 20% of the majority and using the following sample size calculation formula, the sample size was estimated to be 724 people. It was found that 800 people were considered by considering 10% non-cooperation and attrition.

$$n = \frac{{{Z_{1 - \frac{\alpha }{2}}}^2pq}}{{{d^2}}}$$

Quantitative data are represented as mean and standard deviation and qualitative data are shown as frequency and number. Independent sample t-test and Chi-squared test were applied for comparing subjects according to emotional divorce and the odds ratio obtained from univariate logistic regression are reported. Multiple logistic regression was used to determine independent factors affecting emotional divorce. The significance level was set at 0.05 and R 4.0.2 was used for data analysis.

## Results

In this study, 900 people were studied, of which 50% were women and 50% were men. The mean age of them was 39.47 years with a standard deviation of 9.33 years (the age range of study participants was 19–70 years). The mean marriage duration of the people in this study was 12.22 ± 9.32 years. It was observed that in the majority of cases (79.8%), the man was older than the woman. Most of the participants had a bachelor’s degree (34.9%) and 37.3% of the couples both have a university degree up to a bachelor’s. Among the participants, 35.6% were employees, 29.8% were self-employed, 19.2% were housewives or unemployed, 10.1% were medical staff and 6.2% had other occupations.

**Fig. 1:**
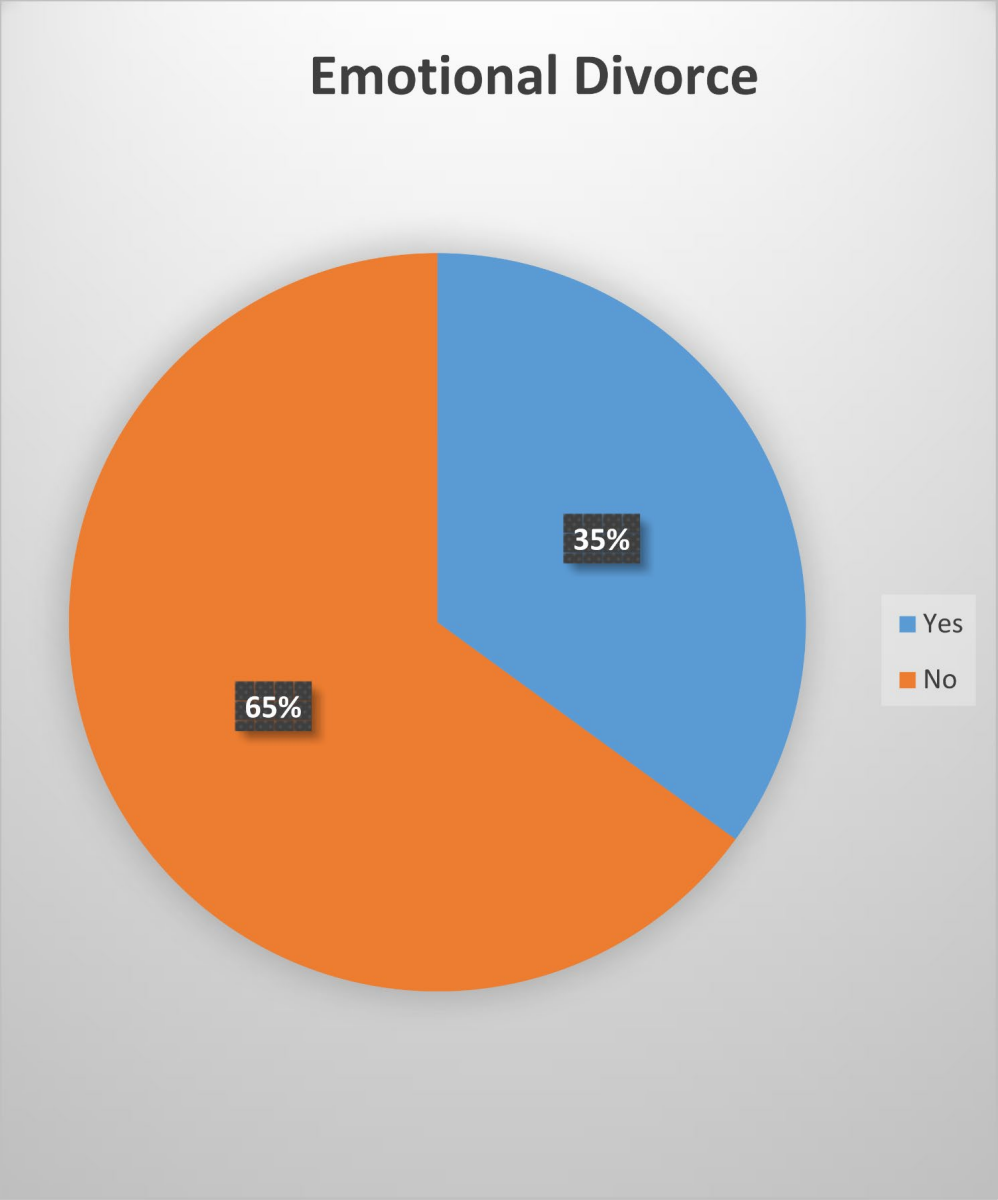
Distribution of this study’s participants in terms of emotional divorce

The characteristics of the participants are reported in Table 1. The mean score of the emotional divorce questionnaire was 7.79 ± 6.64 and the lowest score was zero and the highest score was 24. Of the total study population, 315 (35%) had an emotional divorce (Fig. 1).

**Table 1:** Study subjects’ characteristics

Variable	N (%)	Variable		N (%)	
Age mean ± SD	39.47 ± 9.33	**1st Marriage**			
Marital age mean ± SD	27.26 ± 6.32	Yes		834 (92.7)	
Marriage duration mean ± SD	12.22 ± 9.32	No		66 (7.3)	
**gender**		**Someone lives with us except our children**			
Male	450 (50)	Yes		561 (62.3)	
female	450 (50)	No		339 (37.7)	
**Couple’s Age difference**		**Disability or chronic disease of one the spouse**			
Older	718 (79.8)	Yes		856 (95.1)	
Same	126 (14.0)	No		44 (4.9)	
Younger	56 (6.2)	**Having a child or children with a disability or chronic disease**			
**How to choose a spouse**		Yes		877 (97.4)	
By themselves	431 (47.9)	No		23 (2.6)	
By their parents	251 (27.9)	**Disability or chronic disease of the person living with you**			
By friends & relatives	218 (24.2)	Yes		846 (94.0)	
**Education**		No		54 (6.0)	
Undergraduate or illiterate	44 (4.9)	**Covid10 infection among your family members**			
Diploma	144 (16)	Yes		477 (53.0)	
Associate’s degree	197 (21.9)	No		323 (35.9)	
Bachelor’s degree	314 (34.9)	I don’t know		100 (11.1)	
Master’s degree	143 (15.9)	**The severity of Covid19 infection**			
Doctoral Degree	58 (6.4)	Mild		204 (22.7)	
**Couples’ education level**		Chronic		64 (7.1)	
Both couples have no university education or are illiterate	146 (16.2)	Death		55 (6.1)	
One couple has a university degree and the other does not	150 (16.7)	**Self-infection of Covid19**			
Both couples have a university degree or a bachelor’s degree	336 (37.3)	Yes		509 (56.6)	
One couple has a bachelor’s degree or less and the other has a postgraduate degree	138 (15.3)	No		278 (30.9)	
Both couples have a university degree	130 (14.4)	I don’t know		113 (12.6)	
**Occupation**		**Couple’s Difference in Income level between**			
Housewife/Unemployed	173 (19.2)	There is no difference		635 (70.6)	
Self-employed	260 (28.9)	My income has drastically decreased		53 (5.9)	
Governmental	320 (35.6)	My income has decreased to some extent		77 (8.6)	
Medical Staff	91 (10.1)	My income has increased to some extent		13 (1.4)	
Other	56 (6.2)	My income has increased dramatically		12 (1.3)	
**Employment status of couples**		It does not apply to me		110 (12.2)	
Both unemployed	8 (0.9)	**Existence of Serious dispute between the couple**			
One of them is the medical staff	124 (13.8)	Yes, Before Covid19 outbreak		187 (20.8)	
One is unemployed and the other is employed in non-medical departments	394 (43.8)	Yes, After Covid19 outbreak		120 (13.3)	
Both work in non-medical departments	374 (41.6)	No		593 (65.9)	
**Type of residence**					
Tenant	331 (36.8)				
Owner	445 (49.4)				
My parents’ house or my wife	124 (13.8)				

In unilabiate analysis, it was observed that the mean age (p = 0.011) and duration of marriage (p = 0.008) were significantly higher for people who had an emotional divorce. A higher proportion of people who had an emotional divorce were in the group where men are older than their wives (p = 0.016). The proportion of people whose parents choose their spouse was statistically higher among those with emotional divorce (p < 0.001). Furthermore, being in the first marriage (p = 0.017), the level of education of participants (p = 0.005), the level of education of the couple (p < 0.001), the job of the participants (p < 0.001), employment of the couple (p = 0.016), being infected by Covid-19 (p < 0.001) and the existence of a serious dispute between couples (p < 0.001) had a statistically significant effect on emotional divorce. There is no statistically significant relationship between emotional divorce, age of marriage, gender, type of residence, living with other family members, disability or chronic diseases of one of the spouses or their children, Covid19 infection, and income difference between spouses (p > 0.05 for all comparisons). The results are shown in Table 2.

**Table 2:** Comparison of subjects’ characteristics according to emotional divorce

Variable	Emotional differences		P value	OR (95% CI)
	No (n = 585)	Yes (n = 315)		
	N (%)	N (%)		
**Age; mean ± SD**	38.68 ± 8.86	40.93 ± 10.00	0.001	1.03 (1.01–1.04)
**Marriage age; mean ± SD**	27.05 ± 5.33	27.65 ± 7.81	0. 174	1.02 (0.993–1.04)
**Marriage duration; median (IQR)**	10 (4-170	11 (5–20)	0.008	1.02 (1.01–1.03)
**Gender**			0.295	
Man	300 (51.3)	150 (47.6)		
Woman	285 (48.7)	165 (52.4)		1.16 (0.880–1.2)
**The age difference of Man from his wife**			0.016	
Men are older	452 (77.3)	266 (84.4)		
The same	96 (16.4)	30 (9.5)		0.531 (0.343–0.822)
Woman is older	37 (6.3)	19 (6.0)		0.873 (0.492–1.55)
**Type of choosing a spouse**			< 0.001	
By themselves	358 (61.2)	73 (23.2)		
By Family	93 (15.9)	158 (50.2)		8.33 (5.82–11.93)
By friends or relatives	134 (22.9)	84 (26.7)		3.07 (2.12–4.46)
**1st Marriage**			0.017	
Yes	34 (5.8)	32 (10.2)		
No	551 (94.2)	283 (89.8)		0.546 (0.330–0.903)
**Education Level**			0.005	
Undergraduate or illiterate	19 (3.2 )	25 (7.9)		
Diploma	84 (14.4)	60 (19.0)		0.543 (0.274–1.07)
Associate	117 (20.0)	80 (25.4)		0.520 (0.268–1.01)
Bachelor	234 (40.0)	80 (25.4)		0.260 (0.136–0.497)
Master	107 (18.3)	36 (11.4)		0.256 (0.126–0.518)
Doctorate	24 (4.1)	34 (10.8)		1.08 (0.487–2.38)
**Couples’ education level**			< 0.001	
Both couples have no university education or are illiterate	78 (13.3)	68 (21.6)		
One couple has a university degree and the other does not	97 (16.6)	53 (16.8)		0.627 (0.393–0.999)
Both couples have a university degree or a bachelor’s degree	238 (40.7)	98 (31.1)		0.472 (0.316–0.706)
One couple has a bachelor’s degree or less and the other has a postgraduate degree	99 (16.9)	39 (12.4)		0.452 (0.276–0.740)
Both couples have a university degree	73 (12.5)	57 (18.1)		0.896 (0.557–1.44)
**Occupation**			< 0.001	
Housewife/Unemployed	109 (18.6)	64 (20.3)		
Self-employed	199 (34.0)	61 (19.4)		0.522 (0.343–0.795)
Governmental	189 (32.3)	131 (41.6)		1.18 (0.807–1.73)
Medical Staff	51 (8.7)	40 (12.7)		1.34 (0.797–2.24)
Other	37 (6.3)	19 (6.0)		0.875 (0.464–1.65)
**Employment status of couples**			0.016	
Both work in non-medical departments	246 (42.1)	128 (40.6)		
One is unemployed and the other is employed in non-medical departments	269 (46.0)	125 (39.7)		0.893 (0.661–1.21)
One of them is the medical staff	66 (11.3)	58 (18.4)		1.68 (1.12–2.55)
Both unemployed	4 (1.3)	4 (0.7)		1.92 (0.473–7.81)
**Type of residence**			0.304	
Tenant	219 (37.4)	112 (35.6)		
Owner	293 (50.1)	152 (48.3)		1.01 (0.751–1.37)
My parents or my spouse’s parents’ house	73 (12/5)	51 (16/2)		1.37 (0.894–2.09)
**Someone lives with us except our children**			0.702	
Yes	362 (61/9)	199 (63/2)		
No	116 (36/8)	223 (38/1)		0.946 (0/713-1/26)
**Disability or chronic disease of one the spouse**			0.070	
Yes	562 (96/1)	294 (93/3)		
No	23 (3/9)	21 (6/7)		1.75 (0/950- 3/21)
**Having a child or children with a disability or chronic disease**			0.080	
No	574 (98/1)	303 (96/2)		
Yes	11 (1/9)	12 (3/8)		2.070 (0/901-4/74)
**Disability or chronic disease of the person living with you**			0.746	
No	551 (94/2)	295 (93/7)		
Yes	34 (5/8)	20 (6/3)		1.10 (0/621-1/94)
**Covid10 infection among your family members**			< 0.001	
No	302 (51/6)	175 (55/6)		
I don’t know	63 (10/8)	37 (11/7)		1.01 (0/648-1/58)
Mild	157 (26/8)	47 (14/9)		0.517 (0/355-0/752)
Chronic	31 (5/3)	33 (10/5)		1.84 (1/09 − 3/10)
Death	32 (5/5)	23 (7/3)		1.24 (0/703-2/19)
**Self-infection of Covid19**			0.921	
No	328 (56/1)	181 (57/5)		
Yes	183 (31/3)	95 (30/2)		0.941 (0/692-1/28)
I don’t know	74 (12/6)	39 (12/4)		0.955 (0/622-1/47)
**Couple’s Difference in Income level between**			0.104	
My income has drastically decreased	36 (6/2)	17 (5/4)		
My income has decreased to some extent	61 (10/4)	16 (5/1)		0.555 (0/250-1/23)
There is no difference	400 (68/4)	235 (74/6)		1.24 (0/684-2/26)
My income has increased to some extent	10 (1/7)	3 (1/0)		0.635 (0/155-2/61)
My income has increased dramatically	8 (1/4)	4 (1/3)		1.06 (0/280-4/01)
It does not apply to me	70 (12/0)	40 (12/7)		1.21 (0/604-2/43)
**Existence of a Serious dispute between the couple**			< 0.001	
No	508 (86/8)	85 (27/0)		
Yes, Before Covid19 outbreak	32 (5/5)	155 (49/2)		28.95 (18/56 − 45/15)
Yes, After Covid19 outbreak	45 (7/7)	75 (23/8)		9.96 (6/45 − 15/39)

In the multiple logistic regression model, it was observed that the chance of emotional divorce was 8.5 times (95% distance difference: 5.64–12.84 and p < 0.001) more among whose spouses were chosen by their parents compared to those who chose their spouses themselves, and the risk of Emotional divorce among people whose spouses were introduced by their friends was 2.8 times (95% distance estimate: 1.84–4.23 and p < 0.001) more than who chose their spouses themselves. First marriage reduced the risk of emotional divorce by approximately 50% (OR = 0.529.95% CI 0.290–0.964; p = 0.038), and the risk of emotional divorce is lower among self-employed people than among unemployed people (OR = 0.540.95% CI 0.309–0.944; p = 0.030). in addition, the risk of emotional divorce is significantly lower among people with mild Covid19 infection than for those without (OR = 0.433.95% CI 0.280–0.668; p < 0.001). The results are shown in Table 3.

**Table 3:** Multiple logistic regression results for determining factors affecting emotional divorce

Variable	Odds ratio	Distance Estimation (95%)	P-value
Age	0/999	(0/971-1/03)	0/953
Duration of marriage	0/987	(0/959-1/02)	0/390
**Age distance of man from his wife**			
Man is older			
The same age	0/732	(0/436-1/23)	0/237
Woman is older	0/997	(0/514-1/93)	0/992
**Type of choosing a spouse**			
By themselves			
By Family	**8/51**	**(5/64 − 12/84)**	**>** 0/001
By friends or relatives	**2/79**	**(1/84 − 4/23)**	**>** 0/001
**1st Marriage**			
Yes			
No	**0/529**	**(0/290-0/964)**	**0/038**
**Education Level**			
Undergraduate or illiterate			
Diploma	1/13	(0/507-2/52)	0/764
Associate	2/51	(0/757-8/29)	0/133
Bachelor	1/27	(0/364-4/42)	0/708
Master	0/365	(0/085 − 1/57)	0/175
Doctorate	1/03	(0/204-5/17)	0/973
**Couples’ education level**			
Both couples have no university education or are illiterate			
One couple has a university degree and the other does not	0/435	(0/189-1/01)	0/051
Both couples have a university degree or a bachelor’s degree	0/418	(0/152-1/15)	0/090
One couple has a bachelor’s degree or less and the other has a postgraduate degree	0/702	(0/227-2/18)	0/540
Both couples have a university degree	1/58	(0/403-6/20)	0/511
**Occupation**			
Housewife/Unemployed			
Self-employed	**0/540**	**(0/309-0/944)**	**0/030**
Governmental	0/960	(0/543-1/70)	0/887
Medical Staff	0/586	(0/205-1/69)	0/317
Other	0/571	(0/257-1/27)	0/168
**Employment status of couples**			
Both work in non-medical departments			
One is unemployed and the other is employed in non-medical departments	0/945	(0/175-5/09)	0/947
One of them is the medical staff	0/803	(0/159-4/05)	0/790
Both unemployed	1/22	(0/189-7/90)	0/834
**Covid10 infection among your family members**			
No			
I don’t know	0/919	(0/547-1/55)	0/752
Mild	**0/433**	**(0/280-0/668)**	**>** 0/001
Chronic	1/75	(0/948-3/23)	0/073
Death	1/03	(0/524-2/02)	0/934

To analyze the sensitivity, the results in terms of disagreement with the spouse are shown in Tables 1, 2 and 3 supp. Among those who state that they do not have a serious dispute with their spouse, parental choice of spouse significantly increases the risk of emotional divorce (p < 0.001). However, the risk of emotional divorce is significantly lower among couples who both had postgraduate education (p = 0.004), one couple had a university education (p = 0.008) and both couples had a bachelor’s degree or less (p = 0.031) than couples without a university education or illiterate. Furthermore, the chance of emotional divorce is lower among those who stated Covid19 infection (p = 0.003) or unaware participants (p = 0.023) than those who did not. Among those who stated to have disagreements with their spouse before Covid19 outbreak, the probability of emotional divorce is 84% lower among self-employed than unemployed or homeless ones (p = 0.025). Among those who reported their disagreements after Covid19 outbreak, the chance of emotional divorce is significantly higher among those whose spouses were chosen by their parents than among those who self-selected (p = 0.036). Also, Emotional divorce among those who had mild Covid19 infection (p = 0.003) and who had experienced death caused by Covid19 infection among their family members (p = 0.037) or those who are unaware of their covid-19 infection (p = 0.022) was significantly lower than those who stated that they did not have covid-19 infection among their family members.

## Discussion

According to the findings, one-third of the participants experienced emotional divorce during the Covid-19 period. The factors that can contribute to emotional divorce include age, marital status, duration of marriage, parental choice of spouse, education level of both partners, occupation of the couple, Covid-19 infection among family members, and having a previous dispute with the spouse before the pandemic. According to this study, the rate of emotional divorce during the Covid-19 period is approximately 35%. However, Jay et al. reported a divorce rate of 10–20% during the same period. It’s important to note that the rate of legal divorce is typically lower than emotional divorce since not all emotional divorces result in a legal divorce. Furthermore, this study stated that interactions and social support can reduce the rate of emotional divorce. As well as, the findings of the present study mention the couple’s occupation and education level as effective factors [20, 21]. According to a study analyzing data from five different regions in the United States of America, the rate of divorce initially decreased during the beginning of the pandemic. However, as the duration of the pandemic increased, the rate of declining divorces slowed down [22]. Perhaps the discrepancy can be attributed to individuals being afraid to venture outside due to the quarantine measures imposed during the pandemic. A study conducted in 2018 on the population of Iranian nurses reported the rate of emotional divorce was 7.6% [5]. It is necessary to be mentioned that the difference in this study is probably related to the target population and non-pandemic conditions.

The same as the previous studies, the existence of disputes between couples before the Covid-19 outbreak can be a predictor of emotional divorce during this pandemic [20, 23, 24]. It is important to note that the disputes between the couples before Covid-19 show the challenging dynamics of in couple’s relationship, thus, in quarantine conditions, problems between them could easily be escalated.

Covid-19 infection among family members can increase the tension between couples by affecting the rate of anxiety and increasing the likelihood of emotional divorce [25]. This factor can be exacerbated by worries about losing their jobs and worrying about the infection of their loved ones. While in a study conducted in 2018 in Iran, it was reported that age, duration of marriage, and occupation have no significant effect on emotional divorce [5]. Given that the present study was conducted during the pandemic situation the loss of job and income significantly creates tension between couples. The findings from this quantitative study align with those of qualitative studies conducted within the same cultural context [10, 13].

The results of this present study showed that marriage rank is one of the predictors of emotional divorce. Hence, that marriage multiplicity increases the chances of emotional divorce, which is consistent with studies that have shown psychological disorders as an important factor in this regard [26, 27]. It is necessary to be mentioned that marital disputes and conflicts can be both the cause and effect of divorce. Therefore, couples who remarry may bring their psychological and emotional problems into a new life, so the possibility of re-separation increases, especially in times of crisis.

Overall, this study shows an increasing chance of emotional divorce by family-chosen spouses. This finding is the same as the results of a study that worked on the socio-demographic factors affecting divorce, according to which factors related to parents and family structure can be predictors of marital divorce [28]. Therefore, it seems family chosen spouse can be a sign of the type of family structure and lack of decision-making independence, where the combination of these factors will increase marital conflicts and subsequently the chances of emotional divorce.

In summary, this study suggests that couples who experienced conflicts in their relationship before the pandemic are more susceptible to its impacts. It is important to note that a crisis such as Covid-19 alone cannot cause harm to the relationship between couples. These findings are consistent with the results of Mikaeli’s study, which reported emotional divorce with psychological flexibility, adaptive motivation structure, and communication pattern of dialogue have a negative and significant correlation [27].

The strengths of the present study were the significant sample size and the use of advanced analytics for better control of confounders. However, the conditions of the pandemic and lockdowns led to difficult access to samples and failure to use multiple questionnaires to investigate psychological and sexual disorders as important factors affecting emotional divorce. Therefore, we tried to overcome this limitation by using the initial interview with a clinical psychologist and ruling out the mentioned problems. It’s important to note that divorce is a social phenomenon that can vary depending on the culture. As a result, the factors that contribute to it may differ across cultures.

## Conclusion

Emotional divorce is considered as a social harm which could be the prelude to legal divorce. Therefore, studying affecting factors in any society can pave the way for culture-based interventions to reduce such social harms.

### Electronic supplementary material

Below is the link to the electronic supplementary material.

Supplementary Material 1

## Data Availability

The datasets used and/or analyzed during the current stare study are available from the corresponding author upon reasonable request.
