# Supplementary material for: The assessment of the related factors of emotional divorce among Iranian people during the Covid-19 pandemic: a descriptive study
Source: BMC Psychol. 2023 Oct 27;11:360. doi: 10.1186/s40359-023-01395-w (PMC10612197; doi:10.1186/s40359-023-01395-w)
Supplement: Supplementary file 1 — Supplementary Material 1 [file 40359_2023_1395_MOESM1_ESM.docx]

**Table S1:** Univariate analysis to determine the factors affecting emotional divorce in terms of deep disputes between couples.

| **Variable** | **There is no deep dispute with the spouse** | | **Formation of deep pre-Covid19 disputes** | | **Formation of deep post-Covid19 disputes** | |
| --- | --- | --- | --- | --- | --- | --- |
|  | **Odds ratio**  **(Distance Estimation 95%)** | **P-value** | **Odds ratio**  **(Distance Estimation 95%)** | **P-value** | **Odds ratio**  **(Distance Estimation 95%)** | **P-value** |
| Age | 1.02 (0/993-1/04) | 0/159 | 0.987 (0/950-1/03) | 0/507 | 1.02 (0/974-1/06) | 0/484 |
| Marriage age | 0.969 (0/927-1/01) | 0/158 | 0.999 (0/952-1/05) | 0/984 | 0.989 (0/937-1/04) | 0/674 |
| Marriage duration | 1.03 (1/01-1/05) | **0/025** | 0.992 (0/956-1/03) | 0/686 | 1.02 (0/981-1/07) | 0/284 |
| **Sex** | | | | | | |
| Man | - |  | - |  | - |  |
| Woman | 0.64 (0/403-1/02) | **0/062** | 1.21 (0/560-2/59) | 0/633 | 1.31 (0/623-2/74) | 0/480 |
| **Age distance of man from his wife** | | | | | | |
| Man is older | - |  |  |  |  |  |
| The same age | 0.311 (0/122-0/794) | **0/015** | 1.32 (0/363-4/76) | 00/676 | 0.463 (0/144-1/49) | 0/196 |
| Woman is older | 1.21 (0/513-2/85) | 0/664 | 0.623 (0/119-3/25) | 0/575 | 0.810 (0/214-3/07) | 0/756 |
| **Type of choosing a spouse** | | | | | | |
| By themselves | - |  | - |  | - |  |
| By Family | 4.91 (2/88-8/39) | **>**0/001 | 1.28 (0/382-4/27) | 0/690 | **2.91 (1/04-8/11)** | **0/042** |
| By friends or relatives | 1.45 (0/736-2/77) | 0/256 | 0.833 (0/237-2/93) | 0/776 | 0.818 (0/338-1/98) | 0/657 |
| **1^st^ Marriage** | | | | | | |
| No | - |  | - |  | - |  |
| Yes | 0.437 (0/211-0/907) | **0/026** | 0.671 (0/145-3/11) | 0/611 | 0.221 (0/026-1/86) | 0/164 |
| **Education Level** | | | | | | |
| Undergraduate or illiterate | - |  | - |  | - |  |
| Diploma | 1.15 (0/380-3/49) | 0/804 | 0.223 (0/025-1/99) | 0/179 | 0.271 (0/026-2/78) | 0/271 |
| Associate | 0.782 (0/262-2/34) | 0/660 | 0.258 (0/030-2/24) | 0/219 | 0.667 (0/065-6/79) | 0/732 |
| Bachelor | 0.351 (0/118-1/04) | **0/060** | 0.438 (0/049-3/92) | 0/460 | 0.267 (0/027-2/66) | 0/260 |
| Master | 0.315 (0/094-1/06) | 0/061 | 0.313 (0/029-3/34) | 0/336 | 0.429 (0/040-4/64) | 0/486 |
| Doctorate | 0.850 (0/193-3/74) | 0/830 | 0.214 (0/024-1/91) | 0/168 | 1.50 (0/071-31/58) | 0/794 |
| **Couples' education level** | | | | | | |
| Both couples have no university education or are illiterate | - |  | - |  | - |  |
| One couple has a university degree and the other does not | 0.526 (0/256-1/08) | 0/080 | 0.719 (0/215-2/41) | 0/592 | 0.982 (0/290-3/33) | 0/977 |
| Both couples have a university degree or a bachelor's degree | 0.277 (0/145-0/530) | **>**0/001 | 1.19 (0/375-3/74) | 0/772 | 1.21 (0/435-3/34) | 0/720 |
| One couple has a bachelor's degree or less and the other has a postgraduate degree | 0.454 (0/220-0/940) | 0/033 | 0.710 (0/151-3/33) | 0/664 | 1.33 (0/389-4/52) | 0/651 |
| Both couples have a university degree | 0.398 (0/170-0/928) | 0/033 | 0.919 (0/288-2/93) | 0/887 | 1.79 (0/434-7/36) | 0/422 |
| **Occupation** | | | | | | |
| Housewife/Unemployed | - |  | - |  | - |  |
| Self-employed | 0.897 (0/407-1/98) | 0/787 | **0.157 (0/031-0/792)** | **0/025** | 0.769 (0/272-2/18) | 0/621 |
| Governmental | 2.02 (0/983-4/16) | 0/056 | 0.243 (0/053-1/12) | 0/069 | 0.954 (0/358-2/54) | 0/925 |
| Medical Staff | 2.74 (1/16-6/52) | **0/022** | 0.324 (0/055-1/92) | 0/214 | 1.05 (0/164-6/78) | 0/957 |
| Other | 1.65 (0/566-4/83) | 0/358 | 0.529 (0/043-6/52) | 0/620 | 0.526 (0/108-2/56) | 0/427 |
| **Employment status of couples** | | | | | | |
| Both unemployed | - |  | - |  | -- |  |
| One of them is the medical staff | 1.33 (0/124-12/19) | 0/803 | 1.38 (0/115-12/19) | 0/604 | 2.33 (0/144-21/15) | 0/705 |
| One is unemployed and the other is employed in non-medical departments | 1.68 (0/892-3/15) | 0/109 | 1.05 (0/384-2/89) | 0/921 | 3.91 (0/784-19/46) | 0/096 |
| Both work in non-medical departments | 0.597 (0/353-1/01) | 0/054 | 1.50 (0/630-3/55) | 0/361 | 1.35 (0/616-2/94) | 0/457 |
| **Type of residence** | | | | | | |
| Tenant | - |  | -- |  | - |  |
| Owner | 0.832 (0/514-1/35) | 0/455 | 1.02 (0/429-2/44) | 0/957 | 0.971 (0/425-2/22) | 0/945 |
| My parents or my spouse’s parents’ house | 0.630 (0/267-1/49) | 0/291 | 1.10 (0/363-3/33) | 0/867 | 0.825 (0/289-2/36) | 0/720 |
| **Someone lives with us except our children** | | | | | | |
| No |  |  | -- |  | - |  |
| Yes | **0.457 (0/264-0/793)** | **0/005** | 1.06 (0/487-2/29) | 0/891 | 0.821 (0/391-1/72) | 0/603 |
| **Disability or chronic disease of one the spouse** | | | | | | |
| No | - |  | - |  | - |  |
| Yes | 2.19 (0/839-5/73) | 0/109 | 0.333 (0/104-1/07) | 0/065 | 3.83 (0/445-32/86) | 0/221 |
| **Having a child or children with a disability or chronic disease** | | | | | | |
| No | - |  | - |  | -- |  |
| Yes | 1.51 (0/314-7/22) | 0/608 | 0.397 (0/070-2/27) | 0/299 | 3.83 (0/445-32/86) | 0/221 |
| **Disability or chronic disease of the person living with you** | | | | | | |
| No | - |  | - |  | - |  |
| Yes | 0.508 (0/118-2/20) | 0/365 | 0.738 (0/194-2/81) | 0/657 | 0.476 (0/160-1/42) | 0/182 |
| **Covid10 infection among your family members** | | | | | | |
| No | - |  | - |  | - |  |
| Mild | **0.513 (0/270-0/975)** | **0/042** | 0.568 (0/216-1/50) | 0/251 | **0.192 (0/069-0/534)** | **0/002** |
| Chronic | **2.24 (1/04-4/81)** | **0/039** | 1.12 (0/293-4/28) | 0/869 | 0.655 (0/112-3/84) | 0/655 |
| Death | 0.220 (0/029-1/67) | 0/143 | 1.12 (0/293-4/28) | 0/869 | 0.262 (0/064-1/07) | 0/062 |
| I don’t know | 0.863 (0/386-1/93) | 0/720 | 1.68 (0/353-7/99) | 0/515 | **0.262 (0/093-0/741)** | **0/012** |
| **Self-infection of Covid19** | | | | | | |
| No | - |  | -- |  | - |  |
| Yes | **0.371 (0/198-0/694)** | **0/002** | 0.718 (0/316-1/63) | 0/428 | 1.32 (0/577-2/99) | 0/515 |
| I don’t know | 0.435 (0/180-1/05) | 0/064 | 1.01 (0/303-3/34) | 0/992 | 0.778 (0/276-2/19) | 0/635 |
| **Couple’s Difference in Income level between** | | | | | | |
| My income has drastically decreased | - |  | - |  | -- |  |
| My income has decreased to some extent | 0.497 (0/152-1/62) | 0/247 | 0.280 (0/025-3/20) | 0/305 | 0.600 (0/097-3/72) | 0/097 |
| There is no difference | 0.704 (0/295-1/68) | 0/429 | 1.32 (0/146-11/98) | 0/804 | 2.31 (0/652-8/24) | 0/652 |
| My income has increased to some extent | 0.921 (0/162-5/25) | 0/926 | 0.133 (0/008-2/18) | 0/158 | 1.20 (0/059-24/47) | 0/059 |
| My income has increased dramatically | 1.014 (0/100-10/77) | 0/977 | 0.880 (0/083-9/29) | 0/915 | 1.20 (0/059-24/47) | 0/059 |
| It does not apply to me | 0.592 (0/201-1/75) | 0/342 | 0.501 (0/304-1/21) | 0/142 | 5.40 (0/778-37/51) | 0/778 |

**Table S2:** The results of multiple logistic regression analysis to determine the factors affecting emotional divorce among participants who stated that there was no deep dispute between them and their spouses.

| **Variable** | **Odds ratio (Distance Estimation 95%)** | **P-value** |
| --- | --- | --- |
| Marriage duration | 0.987 (0/956-1/02) | 0/425 |
| **Age distance of man from his wife** | | |
| Man is older | - |  |
| The same age | 0.419 (0/152-1/15) | 0/092 |
| Woman is older | 1.08 (0/411-2/83) | 0/878 |
| **Type of choosing a spouse** | | |
| By themselves | - |  |
| By Family | 4.31 (2/26-8/21) | **>**0/001 |
| By friends or relatives | 1.31 (0/631-2/73) | 0/466 |
| **1^st^ Marriage** | | |
| No | - |  |
| Yes | 0.472 (0/203-1/10) | 0/082 |
| **Couples' education level** | | |
| Both couples have no university education or are illiterate | - |  |
| One couple has a university degree and the other does not | 0.547 (0/237-1/26) | 0/157 |
| Both couples have a university degree or a bachelor's degree | **0.340 (0/153-0/755)** | **0/008** |
| One couple has a bachelor's degree or less and the other has a postgraduate degree | **0.373 (0/152-0/915)** | **0/031** |
| Both couples have a university degree | **0.211 (0/074-0/600)** | **0/004** |
| **Occupation** | | |
| Housewife/Unemployed | - |  |
| Self-employed | 0.840 (0/325-2/17) | 0/719 |
| Governmental | 1.47 (0/566-3/84) | 0/427 |
| Medical Staff | 1.23 (0/225-6/74) | 0/811 |
| Other | 1.22 (0/347-4/25) | 0/761 |
| **Employment status of couples** | | |
| Both unemployed | - |  |
| One of them is the medical staff | 2.05 (0/124-33/83) | 0/617 |
| One is unemployed and the other is employed in non-medical departments | 1.06 (0/097-11/65) | 0/960 |
| Both work in non-medical departments | 1.49 (0/122-18/25) | 0/754 |
| **Someone lives with us except our children** | | |
| No | - |  |
| Yes | 0.696 (0/371-1/30) | 0/258 |
| **Covid10 infection among your family members** |  |  |
| No | -- |  |
| Mild | 0.565 (0/262-1/22) | 0/145 |
| Acute | 3.45 (1/41-8/46) | 0/007 |
| Death | 0.442 (0/052-3/76) | 0/455 |
| I don’t know | 1.19 (0/448-3/17) | 0/727 |
| **Self-infection of Covid19** | | |
| No | - |  |
| Yes | **0.314 (0/147-0/675)** | **0/003** |
| I don’t know | **0.296 (0/104-0/845)** | **0/023** |

**Table S3:** The results of multiple logistic regression analysis to determine the factors affecting emotional divorce among those who stated that a deep post-econological difference had formed between them.

| **Variable** | **OR (95% CI)** | **P-value** |
| --- | --- | --- |
| **Type of choosing a spouse** | | |
| By themselves | - |  |
| By Family | **3.35 (1/09-10/36)** | **0/036** |
| By friends or relatives | 1.06 (0/406-2/78) | 0/900 |
| **Covid10 infection among your family members** | | |
| No | - |  |
| Mild | **0.201 (0/070-0/579)** | **0/003** |
| Acute | 0.496 (0/078-3/15) | 0/457 |
| Death | **0.199 (0/044-0/905)** | **0/037** |
| I don’t know | **0.288 (0/099-0/836)** | **0/022** |
